# *catena*-Poly[[(2,2′-bipyridine-κ^2^ *N*,*N*′)copper(I)]-μ-cyanido-κ^2^ *C*:*N*-[(2,2′-bipyridine-κ^2^ *N*,*N*′)copper(I)]-μ-thio­cyanato-κ^2^ *S*:*N*]

**DOI:** 10.1107/S1600536808037756

**Published:** 2008-11-20

**Authors:** Jun Zhao, Wen-Wen Dong, Dong-Sheng Li, Qiu-Fen He

**Affiliations:** a College of Mechanical and Material Engineering, Functional Materials Research Institue, Three Gorges University, Yichang 443002, People’s Republic of China

## Abstract

The title compound, [Cu_2_(CN)(SCN)(C_10_H_8_N_2_)_2_]_*n*_, contains two crystallographically independent Cu^I^ atoms, each in a distorted tetra­hedral geometry. Each Cu atom is coordinated by a bidentate chelating 2,2′-bipyridine ligand. A bridging cyanide anion links the two Cu(2,2′-bipyridine) units to form a binuclear unit. Adjacent binuclear units are connected by a thio­cyanate anion into a one-dimensional helical chain along [010]. The cyanide anion is disordered, with each site occupied by both C and N atoms in an occupancy ratio of 0.61 (5):0.39 (5). The S atom of the thio­cyanate anion is also disordered over two sites, with occupancy factors of 0.61 (3) and 0.39 (3). There are π–π inter­actions between the pyridyl rings of neighbouring chains [centroid–centroid distance = 3.82 (1) Å].

## Related literature

For general background, see: Hibble & Chippindale (2005 ▶); Krautscheid *et al.* (1998 ▶); Ren *et al.* (2001 ▶). For related structures, see: Liu *et al.* (2006 ▶).
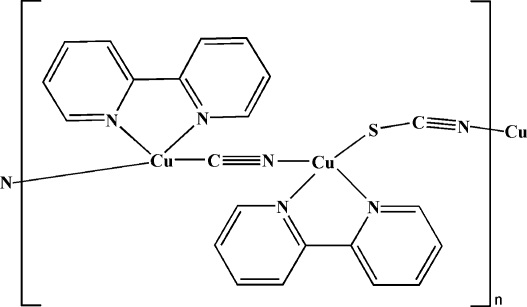


## Experimental

### 

#### Crystal data

[Cu_2_(CN)(SCN)(C_10_H_8_N_2_)_2_]
*M* *_r_* = 523.55
Monoclinic, 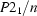

*a* = 14.977 (12) Å
*b* = 9.356 (7) Å
*c* = 17.065 (14) Å
β = 111.532 (12)°
*V* = 2224 (3) Å^3^
*Z* = 4
Mo *K*α radiation
μ = 2.03 mm^−1^
*T* = 293 (2) K
0.45 × 0.12 × 0.10 mm

#### Data collection

Bruker SMART APEX CCD area-detector diffractometer
Absorption correction: multi-scan (*SADABS*; Sheldrick, 1996 ▶) *T*_min_ = 0.521, *T*_max_ = 0.868 (expected range = 0.490–0.817)
16410 measured reflections
5033 independent reflections
3039 reflections with *I* > 2σ(*I*)
*R*_int_ = 0.067

#### Refinement

*R*[*F* ^2^ > 2σ(*F* ^2^)] = 0.058
*wR*(*F* ^2^) = 0.132
*S* = 1.02
5033 reflections
285 parameters
H-atom parameters constrained
Δρ_max_ = 0.77 e Å^−3^
Δρ_min_ = −0.43 e Å^−3^

### 

Data collection: *SMART* (Bruker, 2007 ▶); cell refinement: *SAINT* (Bruker, 2007 ▶); data reduction: *SAINT*; program(s) used to solve structure: *SHELXS97* (Sheldrick, 2008 ▶); program(s) used to refine structure: *SHELXL97* (Sheldrick, 2008 ▶); molecular graphics: *SHELXTL* (Sheldrick, 2008 ▶) and *DIAMOND* (Brandenburg, 1999 ▶); software used to prepare material for publication: *SHELXTL*.

## Supplementary Material

Crystal structure: contains datablocks I, global. DOI: 10.1107/S1600536808037756/hy2162sup1.cif

Structure factors: contains datablocks I. DOI: 10.1107/S1600536808037756/hy2162Isup2.hkl

Additional supplementary materials:  crystallographic information; 3D view; checkCIF report

## Figures and Tables

**Table 1:** Selected bond lengths (Å)

Cu1—C21*A*	1.903 (5)
Cu1—N6^i^	1.964 (5)
Cu1—N1	2.111 (4)
Cu1—N2	2.119 (4)
Cu2—N5*A*	1.889 (5)
Cu2—N3	2.091 (4)
Cu2—N4	2.094 (4)
Cu2—S1*A*	2.465 (15)
Cu2—S1*B*	2.33 (2)

Symmetry code: (i) 
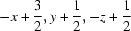
.

**Fig. 1.**
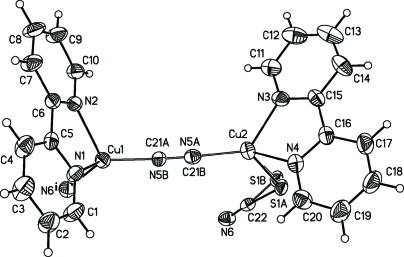
Part of the polymeric structure of the title compound. Displacement ellipsoids are drawn at the 30% probability level. [Symmetry code: (i) -x+3/2, y+1/2, -z+1/2.]

**Fig. 2.**
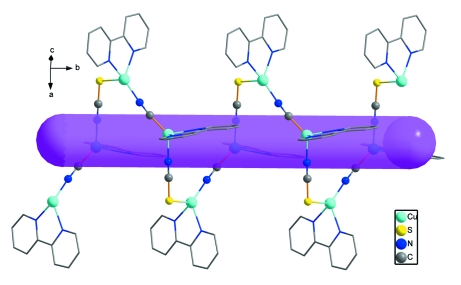
One-dimensional helical chain in the title compound.

## supplementary crystallographic information

### Comment

Transition-metal cyanide or thiocyanate complexes have recently attracted much interest because they can be used as linear linkers in crystal engineering. With their ambidexterous characters the SCN^-^and CN^-^ anions are expected to be involved in a variety of coordination complexes. The syntheses and crystal structures of the complexes of CuSCN and CuCN with various donor ligands like substituted pyridines have been fully investigated (Hibble & Chippindale, 2005; Krautscheid *et al.*, 1998; Ren *et al.*, 2001). However, only a few complexes containing both SCN^-^ and CN^-^ anions have been reported recently (Liu *et al.*, 2006). In this paper, we report the hydrothermal synthesis and structure of a new one-dimensional helical chain formed by both thiocyanate and cyanide anions.

The title compound contains a binuclear unit consisting of two Cu^I^ atoms bridged by a cyanide anion. Each Cu atom is coordinated by a bidentate chelating 2,2'-bipyridine (2,2'-bipy) molecule (Fig. 1). Both Cu^I^ atoms have a distorted tetrahedral geometry (Table 1). The bidentate SCN^-^ ligand links adjacent binuclear [Cu_2_(2,2'-bpy)_2_(CN)] units into a one-dimensional helical chain running along the *b* axis. The intrachain Cu···Cu distance across the cyanide bridge is 4.9263 (3) Å. The helical chain is decorated by 2,2'-bipy ligands towards the lateral of the chain (Fig. 2). There are π–π interactions between the pyridyl rings of neighboring chains [centroid–centroid distance = 3.82 (1)Å]. The cyanide anion is disordered with each site occupied by both C and N atoms in an occupacy ratio of 0.61 (5):0.39 (5). The S atom of the thiocyanate anion is also disordered over two sites with occupacy factors of 0.61 (3) and 0.39 (3).

### Experimental

All chemicals were of reagent grade quality obtained from commercial sources and used without further purification. A mixture of CuSCN (0.07 g, 0.60 mmol), NaCN (0.05 g, 1 mmol), 2,2'-bipy (0.06 g, 0.40 mmol) and water (10 ml) in a 25 ml Teflon-lined stainless steel reactor was heated from 298 to 453 K in 2 h and maintained at 453 K for 72 h. After the mixture was cooled to 298 K, red crystals of the title compound were obtained (yield 45%). IR (KBr pellet, cm^-1^): 3434(m), 2102(s), 1592(m), 1467(m), 1437(s), 1152(w), 759(s), 735(m).

### Refinement

H atoms were positioned geometrically and refined using a riding model, with C—H = 0.93 Å and *U*_iso_(H) = 1.2*U*_eq_(C).

### Crystal data

**Table:**

[Cu_2_(CN)(SCN)(C_10_H_8_N_2_)_2_]	*F*_000_ = 1056
*M**_r_* = 523.55	*D*_x_ = 1.563 Mg m^−^^3^
Monoclinic, *P*2_1_/*n*	Mo *K*α radiation λ = 0.71073 Å
Hall symbol: -P 2yn	Cell parameters from 1688 reflections
*a* = 14.977 (12) Å	θ = 2.5–27.5º
*b* = 9.356 (7) Å	µ = 2.03 mm^−^^1^
*c* = 17.065 (14) Å	*T* = 293 (2) K
β = 111.532 (12)º	Prism, red
*V* = 2224 (3) Å^3^	0.45 × 0.12 × 0.10 mm
*Z* = 4	

### Data collection

**Table:**

Bruker SMART APEX CCD area-detector diffractometer	5033 independent reflections
Radiation source: fine-focus sealed tube	3039 reflections with *I* > 2σ(*I*)
Monochromator: graphite	*R*_int_ = 0.067
*T* = 293(2) K	θ_max_ = 27.4º
φ and ω scans	θ_min_ = 2.5º
Absorption correction: multi-scan(SADABS; Sheldrick, 1996)	*h* = −19→19
*T*_min_ = 0.521, *T*_max_ = 0.868	*k* = −12→11
16410 measured reflections	*l* = −19→22

### Refinement

**Table:**

Refinement on *F*^2^	Secondary atom site location: difference Fourier map
Least-squares matrix: full	Hydrogen site location: inferred from neighbouring sites
*R*[*F*^2^ > 2σ(*F*^2^)] = 0.058	H-atom parameters constrained
*wR*(*F*^2^) = 0.132	*w* = 1/[σ^2^(*F*_o_^2^) + (0.0502*P*)^2^] where *P* = (*F*_o_^2^ + 2*F*_c_^2^)/3
*S* = 1.02	(Δ/σ)_max_ = 0.001
5033 reflections	Δρ_max_ = 0.77 e Å^−^^3^
285 parameters	Δρ_min_ = −0.43 e Å^−^^3^
Primary atom site location: structure-invariant direct methods	Extinction correction: none

### Fractional atomic coordinates and isotropic or equivalent isotropic displacement parameters (Å^2^)

**Table:**

	*x*	*y*	*z*	*U*_iso_*/*U*_eq_	Occ. (<1)
Cu1	0.67204 (4)	0.70063 (6)	0.09388 (4)	0.04969 (19)	
Cu2	0.94638 (4)	0.38679 (6)	0.12252 (4)	0.0539 (2)	
S1A	1.0245 (11)	0.2228 (17)	0.2410 (10)	0.0513 (17)	0.61 (3)
S1B	1.0036 (11)	0.209 (3)	0.2245 (12)	0.0513 (17)	0.39 (3)
N6	0.9046 (3)	0.2050 (4)	0.3342 (3)	0.0567 (11)	
C22	0.9503 (3)	0.2109 (5)	0.2928 (3)	0.0436 (11)	
N1	0.6847 (3)	0.9214 (4)	0.0748 (3)	0.0497 (10)	
N2	0.5776 (3)	0.7276 (4)	−0.0328 (2)	0.0432 (9)	
N3	0.9649 (3)	0.2707 (4)	0.0249 (3)	0.0541 (10)	
N4	1.0819 (3)	0.4562 (4)	0.1291 (3)	0.0495 (10)	
C1	0.7345 (4)	1.0173 (6)	0.1319 (4)	0.0710 (16)	
H1	0.7737	0.9839	0.1847	0.085*	
C2	0.7316 (4)	1.1623 (6)	0.1179 (4)	0.0701 (17)	
H2	0.7671	1.2249	0.1602	0.084*	
C3	0.6755 (4)	1.2114 (6)	0.0408 (4)	0.0769 (18)	
H3	0.6732	1.3086	0.0287	0.092*	
C4	0.6225 (4)	1.1165 (5)	−0.0190 (4)	0.0633 (15)	
H4	0.5825	1.1493	−0.0716	0.076*	
C5	0.6282 (3)	0.9721 (5)	−0.0015 (3)	0.0448 (11)	
C6	0.5712 (3)	0.8629 (5)	−0.0623 (3)	0.0437 (11)	
C7	0.5128 (4)	0.8949 (6)	−0.1445 (3)	0.0662 (15)	
H7	0.5093	0.9882	−0.1642	0.079*	
C8	0.4604 (4)	0.7896 (6)	−0.1966 (3)	0.0787 (18)	
H8	0.4207	0.8107	−0.2516	0.094*	
C9	0.4672 (4)	0.6532 (6)	−0.1670 (4)	0.0708 (17)	
H9	0.4327	0.5794	−0.2013	0.085*	
C10	0.5263 (3)	0.6271 (5)	−0.0849 (3)	0.0526 (12)	
H10	0.5307	0.5339	−0.0649	0.063*	
C11	0.9012 (5)	0.1844 (6)	−0.0297 (4)	0.0720 (16)	
H11	0.8408	0.1758	−0.0264	0.086*	
C12	0.9201 (6)	0.1071 (6)	−0.0909 (4)	0.086 (2)	
H12	0.8737	0.0478	−0.1277	0.103*	
C13	1.0086 (6)	0.1203 (7)	−0.0958 (4)	0.091 (2)	
H13	1.0234	0.0693	−0.1362	0.109*	
C14	1.0758 (5)	0.2089 (6)	−0.0409 (4)	0.0738 (17)	
H14	1.1362	0.2185	−0.0439	0.089*	
C15	1.0525 (4)	0.2834 (5)	0.0187 (3)	0.0510 (12)	
C16	1.1191 (3)	0.3817 (5)	0.0804 (3)	0.0508 (12)	
C17	1.2147 (4)	0.4011 (7)	0.0900 (4)	0.0759 (17)	
H17	1.2407	0.3497	0.0568	0.091*	
C18	1.2706 (4)	0.4969 (8)	0.1488 (4)	0.091 (2)	
H18	1.3342	0.5112	0.1547	0.110*	
C19	1.2339 (4)	0.5700 (7)	0.1979 (4)	0.0810 (19)	
H19	1.2712	0.6339	0.2384	0.097*	
C20	1.1390 (4)	0.5467 (6)	0.1858 (4)	0.0646 (15)	
H20	1.1131	0.5973	0.2194	0.078*	
N5A	0.8391 (3)	0.4961 (4)	0.1195 (3)	0.0479 (14)	0.61 (5)
C21A	0.7751 (3)	0.5696 (4)	0.1130 (3)	0.0426 (13)	0.61 (5)
N5B	0.7751 (3)	0.5696 (4)	0.1130 (3)	0.0426 (13)	0.39 (5)
C21B	0.8391 (3)	0.4961 (4)	0.1195 (3)	0.0479 (14)	0.39 (5)

### Atomic displacement parameters (Å^2^)

**Table:**

	*U*^11^	*U*^22^	*U*^33^	*U*^12^	*U*^13^	*U*^23^
Cu1	0.0544 (4)	0.0509 (4)	0.0433 (4)	0.0116 (3)	0.0172 (3)	0.0052 (3)
Cu2	0.0502 (3)	0.0548 (4)	0.0629 (4)	0.0054 (3)	0.0283 (3)	0.0000 (3)
S1A	0.040 (4)	0.068 (3)	0.045 (4)	0.015 (3)	0.015 (3)	0.014 (3)
S1B	0.040 (4)	0.068 (3)	0.045 (4)	0.015 (3)	0.015 (3)	0.014 (3)
N6	0.059 (3)	0.064 (3)	0.047 (3)	−0.013 (2)	0.020 (2)	−0.003 (2)
C22	0.043 (2)	0.042 (3)	0.038 (3)	−0.003 (2)	0.007 (2)	0.003 (2)
N1	0.044 (2)	0.048 (2)	0.047 (3)	0.0014 (18)	0.0034 (19)	0.0004 (19)
N2	0.050 (2)	0.040 (2)	0.039 (2)	0.0045 (17)	0.0158 (18)	0.0020 (17)
N3	0.067 (3)	0.054 (3)	0.042 (2)	0.004 (2)	0.020 (2)	0.002 (2)
N4	0.051 (2)	0.046 (2)	0.057 (3)	0.0012 (19)	0.025 (2)	0.007 (2)
C1	0.062 (3)	0.060 (4)	0.065 (4)	−0.001 (3)	−0.008 (3)	0.001 (3)
C2	0.056 (3)	0.056 (3)	0.080 (4)	−0.009 (3)	0.003 (3)	−0.010 (3)
C3	0.065 (3)	0.047 (3)	0.098 (5)	−0.005 (3)	0.006 (4)	0.005 (3)
C4	0.059 (3)	0.043 (3)	0.075 (4)	0.003 (2)	0.009 (3)	0.010 (3)
C5	0.037 (2)	0.048 (3)	0.050 (3)	0.006 (2)	0.016 (2)	0.003 (2)
C6	0.046 (2)	0.045 (3)	0.040 (3)	0.007 (2)	0.016 (2)	0.004 (2)
C7	0.092 (4)	0.045 (3)	0.043 (3)	0.007 (3)	0.003 (3)	0.009 (3)
C8	0.101 (4)	0.070 (4)	0.035 (3)	0.011 (3)	−0.010 (3)	0.000 (3)
C9	0.086 (4)	0.052 (3)	0.048 (3)	−0.001 (3)	−0.006 (3)	−0.007 (3)
C10	0.069 (3)	0.038 (3)	0.046 (3)	0.003 (2)	0.015 (3)	0.000 (2)
C11	0.088 (4)	0.065 (4)	0.061 (4)	−0.001 (3)	0.025 (3)	−0.002 (3)
C12	0.130 (6)	0.064 (4)	0.063 (4)	−0.016 (4)	0.034 (4)	−0.010 (3)
C13	0.160 (7)	0.062 (4)	0.069 (5)	−0.004 (4)	0.064 (5)	−0.007 (3)
C14	0.110 (5)	0.059 (4)	0.080 (4)	0.009 (3)	0.066 (4)	0.006 (3)
C15	0.072 (3)	0.045 (3)	0.046 (3)	0.015 (2)	0.033 (3)	0.014 (2)
C16	0.054 (3)	0.054 (3)	0.050 (3)	0.015 (2)	0.026 (3)	0.019 (3)
C17	0.059 (3)	0.113 (5)	0.066 (4)	0.015 (3)	0.035 (3)	0.012 (4)
C18	0.042 (3)	0.151 (7)	0.078 (5)	−0.010 (4)	0.019 (3)	0.002 (5)
C19	0.055 (3)	0.107 (5)	0.081 (5)	−0.015 (3)	0.025 (3)	0.002 (4)
C20	0.069 (3)	0.063 (4)	0.070 (4)	−0.009 (3)	0.036 (3)	−0.005 (3)
N5A	0.052 (3)	0.043 (3)	0.046 (3)	−0.005 (2)	0.014 (2)	−0.002 (2)
C21A	0.043 (3)	0.040 (3)	0.043 (3)	0.004 (2)	0.013 (2)	0.0001 (19)
N5B	0.043 (3)	0.040 (3)	0.043 (3)	0.004 (2)	0.013 (2)	0.0001 (19)
C21B	0.052 (3)	0.043 (3)	0.046 (3)	−0.005 (2)	0.014 (2)	−0.002 (2)

### Geometric parameters (Å, °)

**Table:**

Cu1—C21A	1.903 (5)	C4—H4	0.9300
Cu1—N6^i^	1.964 (5)	C5—C6	1.483 (6)
Cu1—N1	2.111 (4)	C6—C7	1.387 (6)
Cu1—N2	2.119 (4)	C7—C8	1.365 (7)
Cu2—N5A	1.889 (5)	C7—H7	0.9300
Cu2—N3	2.091 (4)	C8—C9	1.363 (7)
Cu2—N4	2.094 (4)	C8—H8	0.9300
Cu2—S1A	2.465 (15)	C9—C10	1.376 (7)
Cu2—S1B	2.33 (2)	C9—H9	0.9300
S1A—C22	1.658 (14)	C10—H10	0.9300
S1B—C22	1.64 (2)	C11—C12	1.382 (8)
N6—C22	1.152 (6)	C11—H11	0.9300
N6—Cu1^ii^	1.964 (4)	C12—C13	1.364 (9)
N1—C1	1.334 (6)	C12—H12	0.9300
N1—C5	1.351 (6)	C13—C14	1.373 (9)
N2—C10	1.329 (6)	C13—H13	0.9300
N2—C6	1.353 (5)	C14—C15	1.379 (7)
N3—C11	1.332 (7)	C14—H14	0.9300
N3—C15	1.359 (6)	C15—C16	1.476 (7)
N4—C20	1.333 (6)	C16—C17	1.392 (7)
N4—C16	1.353 (6)	C17—C18	1.377 (8)
C1—C2	1.375 (7)	C17—H17	0.9300
C1—H1	0.9300	C18—C19	1.344 (8)
C2—C3	1.356 (8)	C18—H18	0.9300
C2—H2	0.9300	C19—C20	1.377 (7)
C3—C4	1.365 (7)	C19—H19	0.9300
C3—H3	0.9300	C20—H20	0.9300
C4—C5	1.380 (6)	N5A—C21A	1.151 (5)
			
C21A—Cu1—N6^i^	121.98 (18)	N2—C6—C7	120.8 (4)
C21A—Cu1—N1	122.94 (16)	N2—C6—C5	116.1 (4)
N6^i^—Cu1—N1	100.50 (16)	C7—C6—C5	123.2 (4)
C21A—Cu1—N2	116.55 (16)	C8—C7—C6	120.1 (5)
N6^i^—Cu1—N2	108.09 (16)	C8—C7—H7	120.0
N1—Cu1—N2	77.86 (14)	C6—C7—H7	120.0
N5A—Cu2—N3	128.31 (17)	C9—C8—C7	119.2 (5)
N5A—Cu2—N4	129.13 (16)	C9—C8—H8	120.4
N3—Cu2—N4	78.14 (17)	C7—C8—H8	120.4
N5A—Cu2—S1B	118.8 (6)	C8—C9—C10	118.4 (5)
N3—Cu2—S1B	96.1 (6)	C8—C9—H9	120.8
N4—Cu2—S1B	95.6 (5)	C10—C9—H9	120.8
N5A—Cu2—S1A	120.1 (3)	N2—C10—C9	123.6 (5)
N3—Cu2—S1A	99.7 (4)	N2—C10—H10	118.2
N4—Cu2—S1A	89.5 (4)	C9—C10—H10	118.2
S1B—Cu2—S1A	7.9 (7)	N3—C11—C12	123.6 (6)
C22—S1A—Cu2	105.7 (7)	N3—C11—H11	118.2
C22—S1B—Cu2	112.6 (11)	C12—C11—H11	118.2
C22—N6—Cu1^ii^	178.3 (4)	C13—C12—C11	118.1 (7)
N6—C22—S1B	172.6 (7)	C13—C12—H12	120.9
N6—C22—S1A	174.8 (7)	C11—C12—H12	120.9
C1—N1—C5	116.9 (4)	C12—C13—C14	119.9 (6)
C1—N1—Cu1	127.1 (4)	C12—C13—H13	120.0
C5—N1—Cu1	115.6 (3)	C14—C13—H13	120.0
C10—N2—C6	117.9 (4)	C13—C14—C15	119.2 (6)
C10—N2—Cu1	127.2 (3)	C13—C14—H14	120.4
C6—N2—Cu1	114.9 (3)	C15—C14—H14	120.4
C11—N3—C15	117.6 (5)	N3—C15—C14	121.7 (5)
C11—N3—Cu2	126.9 (4)	N3—C15—C16	114.7 (4)
C15—N3—Cu2	115.5 (3)	C14—C15—C16	123.6 (5)
C20—N4—C16	118.3 (4)	N4—C16—C17	119.9 (5)
C20—N4—Cu2	125.9 (3)	N4—C16—C15	115.9 (4)
C16—N4—Cu2	114.7 (3)	C17—C16—C15	124.2 (5)
N1—C1—C2	124.3 (5)	C18—C17—C16	119.7 (6)
N1—C1—H1	117.9	C18—C17—H17	120.2
C2—C1—H1	117.9	C16—C17—H17	120.2
C3—C2—C1	118.1 (5)	C19—C18—C17	120.4 (6)
C3—C2—H2	120.9	C19—C18—H18	119.8
C1—C2—H2	120.9	C17—C18—H18	119.8
C2—C3—C4	119.2 (5)	C18—C19—C20	117.6 (6)
C2—C3—H3	120.4	C18—C19—H19	121.2
C4—C3—H3	120.4	C20—C19—H19	121.2
C3—C4—C5	120.2 (5)	N4—C20—C19	124.1 (5)
C3—C4—H4	119.9	N4—C20—H20	117.9
C5—C4—H4	119.9	C19—C20—H20	117.9
N1—C5—C4	121.3 (4)	C21A—N5A—Cu2	174.5 (4)
N1—C5—C6	115.4 (4)	N5A—C21A—Cu1	174.5 (4)
C4—C5—C6	123.3 (4)		
			
N5A—Cu2—S1A—C22	12.1 (10)	C1—N1—C5—C6	−177.9 (4)
N3—Cu2—S1A—C22	−133.7 (7)	Cu1—N1—C5—C6	−4.4 (5)
N4—Cu2—S1A—C22	148.5 (8)	C3—C4—C5—N1	0.9 (8)
S1B—Cu2—S1A—C22	−71 (6)	C3—C4—C5—C6	178.5 (5)
N5A—Cu2—S1B—C22	−7.1 (12)	C10—N2—C6—C7	0.0 (7)
N3—Cu2—S1B—C22	−147.4 (9)	Cu1—N2—C6—C7	179.2 (4)
N4—Cu2—S1B—C22	134.0 (9)	C10—N2—C6—C5	178.8 (4)
S1A—Cu2—S1B—C22	95 (7)	Cu1—N2—C6—C5	−1.9 (5)
Cu2—S1B—C22—S1A	−109 (7)	N1—C5—C6—N2	4.2 (6)
Cu2—S1A—C22—S1B	59 (6)	C4—C5—C6—N2	−173.6 (4)
C21A—Cu1—N1—C1	−70.8 (5)	N1—C5—C6—C7	−177.0 (5)
N6^i^—Cu1—N1—C1	68.9 (4)	C4—C5—C6—C7	5.2 (7)
N2—Cu1—N1—C1	175.3 (5)	N2—C6—C7—C8	0.4 (8)
C21A—Cu1—N1—C5	116.5 (3)	C5—C6—C7—C8	−178.4 (5)
N6^i^—Cu1—N1—C5	−103.8 (3)	C6—C7—C8—C9	−0.6 (9)
N2—Cu1—N1—C5	2.6 (3)	C7—C8—C9—C10	0.5 (9)
C21A—Cu1—N2—C10	58.0 (4)	C6—N2—C10—C9	−0.1 (7)
N6^i^—Cu1—N2—C10	−83.9 (4)	Cu1—N2—C10—C9	−179.3 (4)
N1—Cu1—N2—C10	178.9 (4)	C8—C9—C10—N2	−0.1 (9)
C21A—Cu1—N2—C6	−121.2 (3)	C15—N3—C11—C12	0.7 (8)
N6^i^—Cu1—N2—C6	97.0 (3)	Cu2—N3—C11—C12	−177.6 (4)
N1—Cu1—N2—C6	−0.3 (3)	N3—C11—C12—C13	−0.2 (10)
N5A—Cu2—N3—C11	−44.5 (5)	C11—C12—C13—C14	−0.2 (10)
N4—Cu2—N3—C11	−175.5 (4)	C12—C13—C14—C15	0.1 (9)
S1B—Cu2—N3—C11	90.0 (6)	C11—N3—C15—C14	−0.9 (7)
S1A—Cu2—N3—C11	97.1 (6)	Cu2—N3—C15—C14	177.7 (4)
N5A—Cu2—N3—C15	137.1 (3)	C11—N3—C15—C16	179.4 (4)
N4—Cu2—N3—C15	6.1 (3)	Cu2—N3—C15—C16	−2.1 (5)
S1B—Cu2—N3—C15	−88.4 (5)	C13—C14—C15—N3	0.5 (8)
S1A—Cu2—N3—C15	−81.3 (5)	C13—C14—C15—C16	−179.8 (5)
N5A—Cu2—N4—C20	52.6 (5)	C20—N4—C16—C17	−0.3 (7)
N3—Cu2—N4—C20	−177.2 (4)	Cu2—N4—C16—C17	−169.0 (4)
S1B—Cu2—N4—C20	−82.2 (7)	C20—N4—C16—C15	−179.9 (4)
S1A—Cu2—N4—C20	−77.2 (5)	Cu2—N4—C16—C15	11.4 (5)
N5A—Cu2—N4—C16	−139.7 (3)	N3—C15—C16—N4	−6.2 (6)
N3—Cu2—N4—C16	−9.5 (3)	C14—C15—C16—N4	174.0 (5)
S1B—Cu2—N4—C16	85.5 (7)	N3—C15—C16—C17	174.2 (5)
S1A—Cu2—N4—C16	90.5 (5)	C14—C15—C16—C17	−5.5 (8)
C5—N1—C1—C2	0.0 (8)	N4—C16—C17—C18	−0.3 (8)
Cu1—N1—C1—C2	−172.6 (4)	C15—C16—C17—C18	179.2 (5)
N1—C1—C2—C3	−0.9 (9)	C16—C17—C18—C19	1.0 (10)
C1—C2—C3—C4	1.7 (9)	C17—C18—C19—C20	−1.0 (10)
C2—C3—C4—C5	−1.7 (9)	C16—N4—C20—C19	0.4 (8)
C1—N1—C5—C4	0.0 (7)	Cu2—N4—C20—C19	167.7 (4)
Cu1—N1—C5—C4	173.5 (4)	C18—C19—C20—N4	0.3 (9)

Symmetry codes: (i) −*x*+3/2, *y*+1/2, −*z*+1/2; (ii) −*x*+3/2, *y*−1/2, −*z*+1/2.
